# Immobilization of Olive Leaf Extract with Chitosan Nanoparticles as an Adjunct to Enhance Cytotoxicity

**DOI:** 10.1021/acsomega.3c01494

**Published:** 2023-08-01

**Authors:** Burcu Özdamar, Yusuf Sürmeli, Gülşah Şanlı-Mohamed

**Affiliations:** † Department of Chemistry, İzmir Institute of Technology, 35430 İzmir, Turkey; ‡ Department of Biotechnology and Bioengineering, İzmir Institute of Technology, 35430 İzmir, Turkey; § Department of Agricultural Biotechnology, Namık Kemal University, 59030 Tekirdağ, Turkey

## Abstract

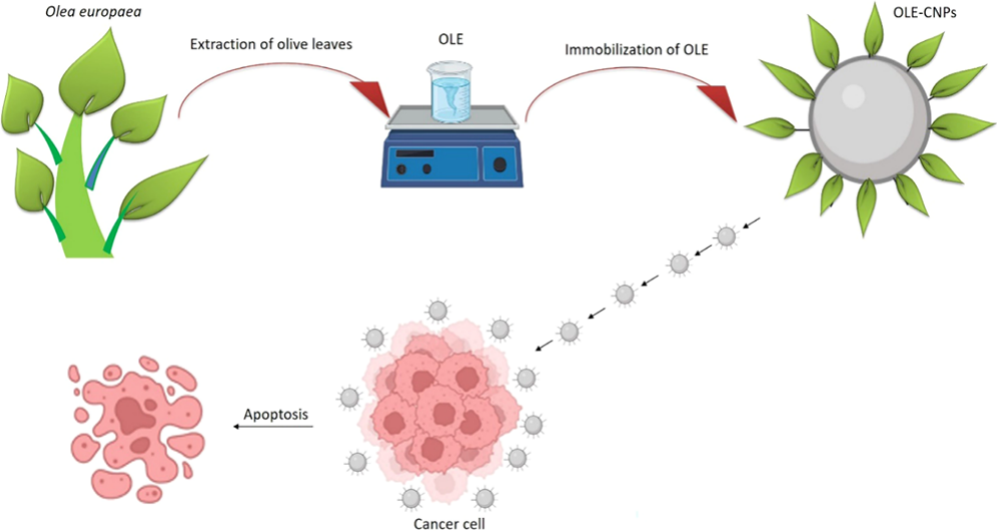

We immobilized the olive leaf extract (OLE) with chitosan nanoparticles (CNPs) by optimizing the effect of various immobilization conditions, and OLE-loaded CNPs (OLE-CNPs) were then elaborately characterized physicochemically by scanning electron microscopy (SEM), Fourier transform infrared (FT-IR) spectroscopy, dynamic light scattering (DLS), and atomic force microscopy (AFM). Under optimal conditions, CNPs were able to accommodate the OLE with a loading capacity of 97.5%. The resulting OLE-CNPs had a spherical morphology, and their average diameter was approximately 100 nm. The cytotoxic influence, cell cycle distribution, and apoptosis stage of OLE and OLE-CNPs were analyzed on lung carcinoma (A549) and breast adenocarcinoma (MCF-7) cell lines. In an in vitro cytotoxic assay, IC_50_ values of OLE-CNPs were determined to be 540 μg/mL for A549 and 810 μg/mL for MCF-7. The treatment of both A549 and MCF-7 with OLE-CNPs caused the highest cell arrest in G0/G1 in a dose-independent manner. OLE-CNPs affected cell cycle distribution in a manner different from free OLE treatment in both cancer cells. A549 and MCF-7 cells were predominantly found in the late apoptosis and necrosis phases, respectively, upon treatment of 1000 μM OLE-CNPs. Our results suggest that CNPs enhance the utility of OLEs as nutraceuticals in cancer and that OLE-CNPs can be utilized as an adjunct to cancer therapy.

## 1 Introduction

Nanotechnology has a wide variety of applications in the medical, industrial, and genetic sciences, and with the use of nanotechnology, the scope of research has radically changed.^1−3^ Nanomaterials used in nanotechnology can have a broad diversity of physical and chemical properties, such as diameters, shapes, and surface structures.^4^ They are utilized in different applications, including anticancer drugs, antimicrobials, and drug delivery.^5−8^

Among nanomaterials, chitosan is a biocompatible and nontoxic material and is largely utilized as a polymer with the ability of nanoparticle formation.^9^ Chitosan is a biopolymer obtained from chitin, which is a structural component of crustacean shells, the cell wall of fungi, and insect cuticles,^10^ and it has a linear polysaccharide structure composed of d-glucosamine and N-acetyl-d-glucosamine residues with the linkage of β-(1–4) glycosidic bonds.^11^ Also, it possesses some beneficial properties (e.g., high stability, biological degradability, and sufficient permeability), which enable the use of chitosan as an ideal biological polymer in medical applications.^12,13^ There have been some recent reports on the use of chitosan-derived nanomaterials in medicine. Accordingly, chitosan nanoparticles have been used to release two antibiotics (metronidazole and sodium ceftriaxone) and to increase the bactericidal strength for the treatment of intra-abdominal infections.^14^ In addition, a carboxymethyl chitosan-coated poly(lactide-*co*-glycolide) (cmcPLGA) core–shell nanostructure caused strong inhibition, even at low concentrations, of A549 (lung adenocarcinoma), PC3 (prostate cancer), and BT549 (breast ductal carcinoma) cell lines.^15^

*Olea europaea*, the olive tree, has held an important place in the herbal medicine of Mediterranean and European countries for many years.^16^ Olive leaves possess a range of therapeutic features, including anticancer, antioxidant, antimicrobial, antiviral (e.g., SARS CoV-2), anti-inflammatory, as well as cardioprotective, neuroprotective, and hepatoprotective activities.^17−19^ Olive leaf extract (OLE) is rich in phenolics such as oleuropein, apigenin, and hydroxytyrosol.^17^ Due to phenolics have an unpleasant taste and odor, it is suggested that they should be covered with another material before their use as oral drugs or supplements in foodstuffs.^20^

There have been few studies on the immobilization of tree leaf extracts used in herbal medicine with chitosan and their effects on cancer cells. The immobilization of the leaf extract of Annona squamosa, custard apple, with chitosan nanoparticles (nano-ASLE) had a cytotoxic effect and induced apoptosis in human colon cancer (WiDr) and HeLa cells.^21,22^ Also, another recent work showed that the immobilization of the leaf extract of Dendrophthoe pentandra, a parasitic plant growing on the mango tree, with chitosan had a cytotoxic effect on the G2/M cell cycle arrest in MCF-7 breast cancer cells.^23^ Besides, the antifungal effect of OLE-immobilized chitosan nanoparticles has been demonstrated in different studies.^24,25^ Regarding these, a recent study has reported the control of verticillium wilt on tomato plants by OLE-loaded CNPs.^25^ However, to the best of our knowledge, no study has been reported about OLE-loaded chitosan nanoparticles (OLE-CNPs) on breast adenocarcinoma (MCF-7) and lung carcinoma (A549) cell lines thus far. In the present work, we immobilized OLE with chitosan nanoparticles and characterized OLE-CNPs by the Folin–Ciocalteu method using an FT-IR spectrometer, AFM, and Zetasizer Nano-ZS. Then, we investigated the influence of OLE-CNPs on lung carcinoma (A549) and breast adenocarcinoma (MCF-7) cell lines.

## 2 Materials and Methods

### 2.1 Materials

Olive leaves were supplied by the Olive Research Institute (İzmir, Turkey). Unless otherwise stated, all chemicals, including chitosan (CS) (medium molecular weight, non-animal-derived, soluble in dilute aqueous acid; viscosity, 200–800 cP) and sodium tripolyphosphate (TPP), were purchased from Sigma.

### 2.2 Extraction of Olive Leaves

Olive leaf extraction was carried out according to the procedure below: olive leaves were cleaned using *d*H_2_O, dried at 37 °C for 72 h, powdered, and extracted with 70% ethanol at 25 °C for 2 h. The liquid-phase, including ethanol, was removed using a vacuum filter and a rotary evaporator at 38 °C and 120 rpm, respectively. The olive leaf extract (OLE) was dried using a freeze-dryer at −52 °C and 0.2 mbar conditions. It was stored in a light-protected bottle.

### 2.3 OLE Characterization

#### 2.3.1 Determination of the Total Phenolic Content

The total phenolic content of OLE was investigated through the Folin–Ciocalteu procedure as explained by Bayçın et al.^26^ In brief, OLE was dissolved in *d*H_2_O, and 20 μL of the sample was added to 100 μL of Folin reagent in a 96-well plate and incubated of 2 min at room temperature (RT). Then, 80 μL of 7% Na_2_CO_3_ was supplemented into the mixture and incubated for 60 min. The spectrophotometric measurement of the samples was performed at OD_725_. This analysis was carried out in triplicate, and the results are expressed as milligrams of gallic acid equivalents per gram of dry olive leaf (mg GAE/g) using a gallic acid calibration curve.

#### 2.3.2 OLE Composition Investigation

The OLE composition was investigated by HPLC analysis using a Hewlett-Packard Series HP 1100 equipped with a diode array detector and a C18 LiChrospher 100 analytical column (250 mm × 4 mm). The flow rate was 1 mL/min, and the absorbance change was monitored at OD_280_. 2.5/97.5 (v/v) of acetic acid/water (A) and acetonitrile (B) were utilized as mobile phases; the linear gradient applied for 60 min was as follows: 20 min from 95% A–5% B to 75% A–25% B, 20 min at 50% A–50% B, and 10 min at 20% A–80% B; finally, re-equilibration of the system was carried out for 10 min, and the system had the initial composition.

The oleuropein content of OLE was also investigated via an HPLC assay utilizing an oleuropein calibration curve. Coumarin was used as an internal standard to measure the antioxidant oleuropein.

#### 2.3.3 Antioxidant Capacity Test

The Trolox equivalent antioxidant capacity (TEAC) test was carried out to investigate the total antioxidant capacity of OLE. For this, sodium persulfate (K_2_S_2_O_4_) was added to a 2,2′-azinobis-(3-ethylbenzothiazoline-6-sulfonic acid) (ABTS+) working solution at an equal amount and then incubated at RT in the dark for 12–16 h. The mixture was spectrophotometrically measured at 734 nm wavelength. The results were expressed as mmol TEAC/g OLE of the sample using a Trolox calibration curve.

### 2.4 Preparation and Optimization of OLE-CNPs

Chitosan nanoparticles were synthesized by the following procedure: 0.5% chitosan dissolved in 1% (w/v) acetic acid solution was incubated at 115 rpm until it was transparent, and the pH was adjusted to 5.0 at room temperature. 0.25% OLE was supplemented into the chitosan solution, and the mixture was incubated for 30 min. Then, the sodium tripolyphosphate (TPP) solution (0.1%) was added to the mixture and stirred at room temperature for 1 h. The mixture was centrifuged at 13500 rpm for 30 min and washed twice with deionized water, and then the freeze-dried nanoparticles were stored at 4 °C for further analyses. The loading capacity of OLE-CNPs was optimized by investigating the effect of various conditions, including TPP concentrations (0.08–3%), pH (4–5), incubation times of TPP (15–120 min) and OLE (15–120 min), and OLE concentration (0.1–2%). The loading capacity of the nanoparticles was calculated using the equation below
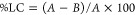
where “*A*” is the total amount of OLE and “*B*” is the amount of free OLE.

### 2.5 Characterization of OLE-CNPs

The loading capacity of nanoparticles was investigated using the Folin–Ciocalteu method by spectrophotometric analysis.^27^ In addition, the morphology of nanoparticles was characterized using Nanomagnetic Instruments ezAFM on tapping mode. Surface morphologies and structures of the samples were analyzed by SEM (Philips XL-30S FEG, Eindhoven, The Netherlands). The nanoparticle size with the size distribution was determined using a Zetasizer Nano-ZS (Malvern Instruments) based on dynamic light scattering (DLS) techniques. Interactions between the chitosan nanoparticles and OLE were analyzed by Fourier transform infrared (FT-IR) spectroscopy according to the Miracle Zn-Se ATR method on a Spectrum-100 FT-IR Spectrometer (Perkin Elmer) in the range of 650–4000 cm^–1^

### 2.6 Influence of OLE-CNPs on Cancer Cell Lines

#### 2.6.1 Cytotoxicity Analysis

In this study, MCF-7 (breast adenocarcinoma), A549 (lung carcinoma), and BEAS-2B (human bronchial epithelium) were used as model cells. Cytotoxicity analyses were carried out to test various concentrations of OLE-CNPs against MCF-7 and A549 by the MTT assay. For this, 1 × 10^4^ cells/mL of each cell line were cultured in 96-well plates and incubated for 24 h. The cells were subjected to a concentration range of 1–1000 μg/mL of OLE and OLE-CNPs. The incubation of the treated and control cells was performed at 37 °C for 72 h. Then, the cultures were washed with PBS buffer, 100 μL of 10% MTT was added to each well, and plates were incubated at 37 °C with 5% CO_2_ for 4 h. The cultures were centrifuged at 1800 rpm for 10 min, and the pellets were dissolved in DMSO. The absorbance of the samples was spectrophotometrically measured at 540 nm wavelength. The cytotoxicity of OLE and OLE-CNPs was evaluated according to the percent cell viability, which is expressed as the ratio of the absorbance of the treated cells to the untreated (control) cells multiplied by 100. “*A*_blank_” refers to the absorbance value of the blank sample, while “*A*_sample_ and *A*_control_” refer to the absorbance values of the actual sample and the control being tested, respectively.
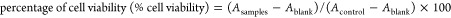
The concentration inhibiting cell viability by 50% (IC_50_) was calculated by a standard curve of cell viability. This assay was carried out in triplicate.

#### 2.6.2 Cell Cycle Assay

The antiproliferative action of free OLE and OLE-CNPs on the cell cycle of MCF-7 and A549 was investigated by flow cytometry with propidium iodide (PI) as the fluorescent stain. For this purpose, A549 and MCF-7 cells were cultured in 6-well plates, including a 1.98 mL growth medium, at a density of 1 × 10^5^ cells/well overnight. Then, 20 μL of free OLE and OLE-CNPs were added into each well in the concentration range of 300–100 μg/mL for A549 and 100–1000 μg/mL for MCF-7 cell lines. The cultures were incubated at 37 °C and 5% CO_2_ conditions for 72 h. The cells were fixed using trypsin, PBS, and cold ethanol, respectively. The fixed cells were harvested at 4 °C and 1200*g* for 10 min by centrifugation. The cell pellets were treated with 200 μL of Triton X-100 (0.1%) and 20 μL of RNase A (200 μg/mL), respectively, and the cell suspensions were then incubated at 37 °C and 5% CO_2_ conditions for 30 min. After the addition of 20 μL of 1 mg/mL PI, the samples were incubated at room temperature for 15 min. The cell cycle distribution was determined by a flow cytometer (FACSCANTO, BD). The obtained data were analyzed by ModFit software and were collected for a minimum of 10.000 events for each sample.

#### 2.6.3 Apoptotic Effect of OLE-CNPs on Cancer Cells

The apoptotic effect of OLE-CNPs on MCF-7 and A549 was investigated using an Annexin V- FITC Detection Kit. In brief, the cancer cells were cultured onto a 6-well plate with a 1.98 mL growth medium at a density of 1 × 10^5^ cells/well and incubated at 37 °C and 5% CO_2_ for 24 h. The cell cultures were then subjected to 20 μL of OLE-CNPs in the concentration range of 100–1000 μg/mL. The treated and control cell cultures were incubated at 37 °C and 5% CO_2_ for 48 h. The cultures were harvested two times at 800 rpm for 5 min, washed with PBS, and the pellets were resuspended in 200 μL binding buffer. 2 μL of Annexin V-FITC and PI were then supplemented, and the stained cells were incubated at room temperature for 15 min, and the mixtures were analyzed using a flow cytometer (FACSCANTO, BD).

#### 2.6.4 Optical Microscopy Display

The influence of free OLE and OLE-CNPs was monitored by optical microscopy. For this purpose, MCF-7 and A549 cell cultures were prepared in a 96-well plate and incubated overnight. Cytotoxic concentrations of free OLE and OLE-CNPs were supplemented into each well, including the MCF-7 or A549 cell culture, and the plates were incubated for 72 h. Finally, the cells were monitored by optical microscopy.

## 3 Results and Discussion

In this work, OLE was immobilized by chitosan nanoparticles, and OLE-CNPs were optimized, characterized, and their influence on cancer cells (breast adenocarcinoma MCF-7 and lung carcinoma A549) and healthy cells (human bronchial epithelium cells BEAS-2B) was investigated. For this, OLE was characterized to determine the total antioxidant capacity, the total phenolic content, and the OLE content. The antioxidant capacity was found to be 2.18 mmol of TEAC/g OLE, and the total phenolic content of OLE was calculated to be 260 mg GAE/g extract. The OLE content analyzed by HPLC (Figure S1) showed that the most prevalent phenolic compound in OLE, among other compounds, was oleuropein, which represented 2.3% (w/v) of OLE. A recent study showed the TPC value of Chinese olive (*O. europaea*) leaves to be 197.32 mg/g of dry matter.^28^ Another study showed that the leaves of 10 major Greek olive varieties possessed a total phenolic content of up to 20 mg GAE/g tissue.^29^

### 3.1 Optimization of OLE-CNPs

**Figure 1:**
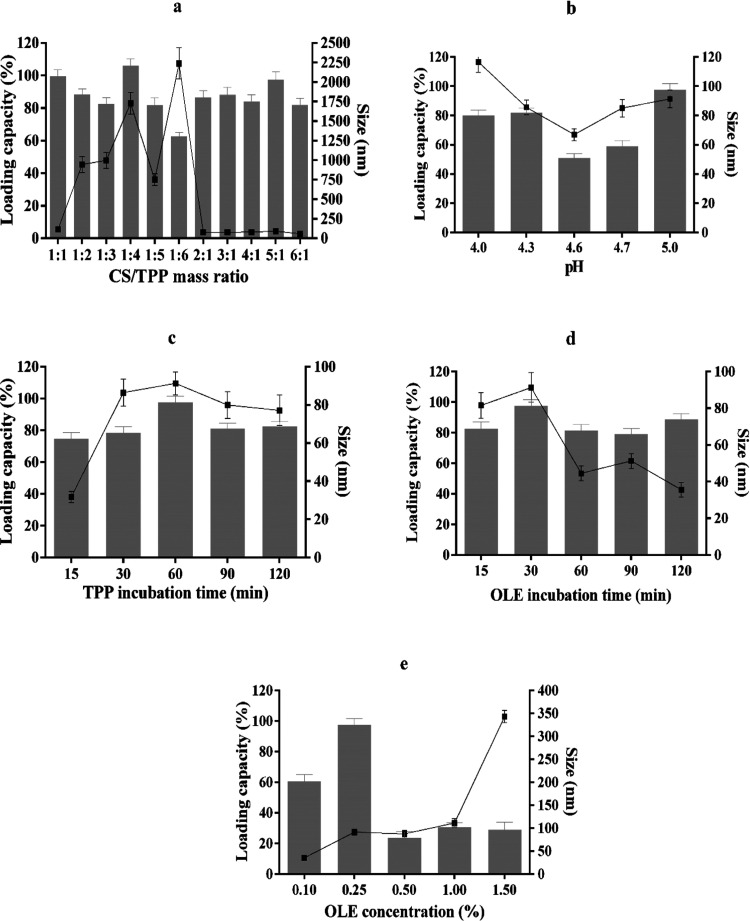
Optimization of the loading capacity and chitosan nanoparticle sizes under various conditions: (a) chitosan/TPP mass ratio; (b) pH of Tris-HCl buffer; (c) incubation time of TPP; (d) incubation time of OLE; and (e) OLE concentration. (lines refer to the nanoparticle size, and columns refer to the loading capacity).

OLE was immobilized by using chitosan nanoparticles, and the loading capacity of OLE-CNPs was optimized by investigating the effect of various conditions, including the chitosan/TPP mass ratio (1:1, 1:2, 1:3, 1:4, 1:5, 1:6, 2:1, 3:1, 4:1, 5:1, and 6:1), pH (4, 4.3, 4.6, 4.7, and 5.0), incubation times of TPP (15, 30, 60, 90, and 120 min) and OLE (15, 30, 60, 90, and 120 min), and the OLE concentration (0.1, 0.25, 0.5, 1.0, and 1.5%). The size distribution of OLE-CNPs was also determined using a Zetasizer Nano-ZS (Malvern Instruments) based on dynamic light scattering (DLS) techniques under the above-mentioned conditions. The analyses showed that the optimal conditions were a 5:1 chitosan/TPP mass ratio, pH 5.0 of Tris-HCl buffer, 60 min incubation time of TPP, 30 min incubation time of OLE, and 0.25% OLE concentration (Figure 1).

### 3.2 Characterization of OLE-CNPs

**Figure 2:**
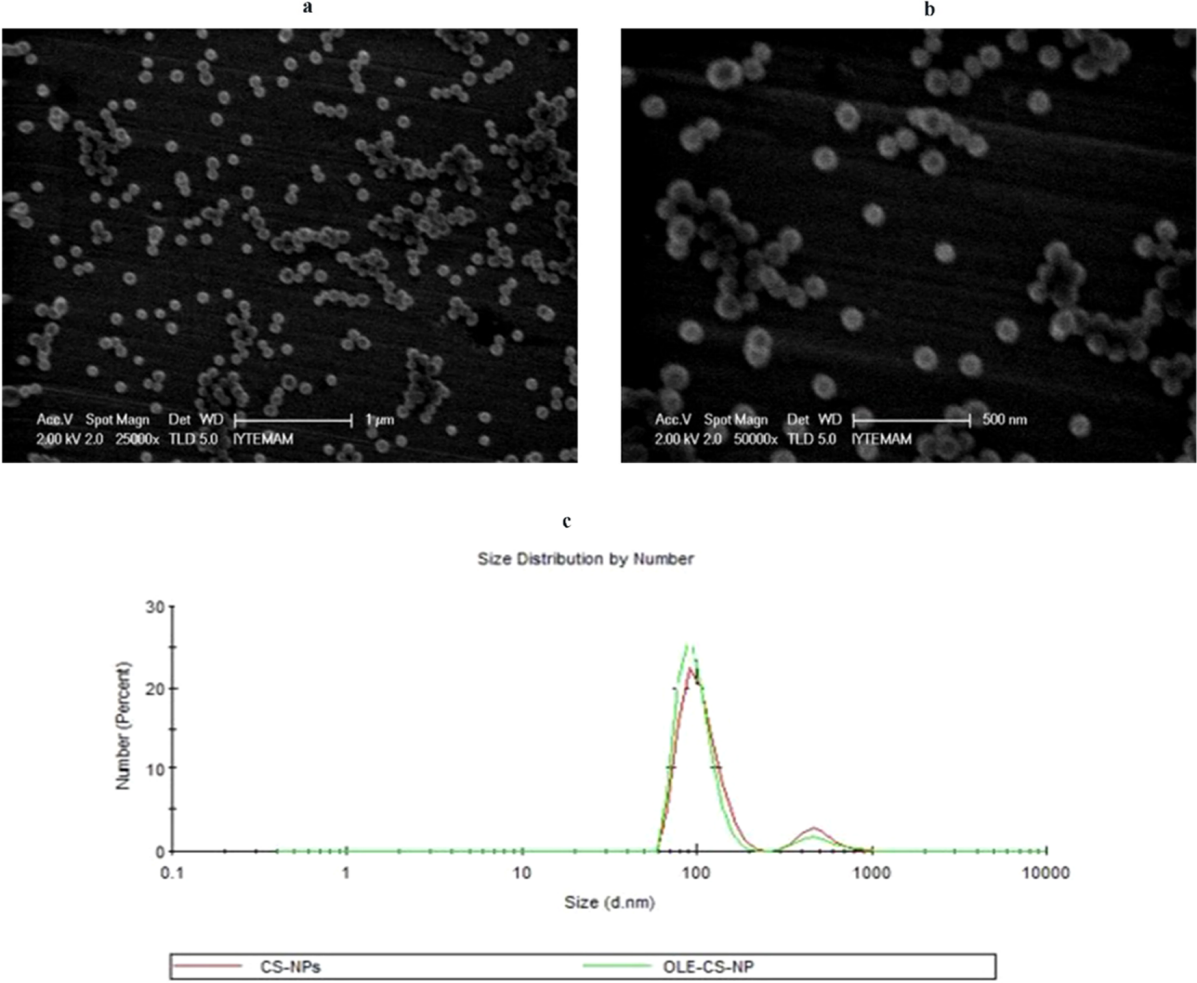
Scanning electron microscopy images of (a) CNPs, (b) OLE-CNPs with different magnifications, and (**c**) size distribution of CNPs and OLE-CNPs by DLS.

OLE-CNPs were characterized in terms of the loading capacity, size, morphology, and interactions between OLE and chitosan nanoparticles. The loading capacity of OLE to CNPs was found to be 97.5% under optimum conditions. The size distribution of OLE-loaded nanoparticles was about 100 nm by dynamic light scattering measurements (Figure 2a,b). The size distribution of OLE-CNPs was very similar to that of CNPs, as illustrated in Figure 2c. This indicated that both types of nanoparticles have a narrow size range, with an average hydrodynamic diameter of around 100 nm. The spherical morphology of both types of nanoparticles was observed by scanning electron microscopy (SEM), which also revealed that they were uniformly dispersed (Figure 2). The nanoparticle sizes observed by SEM were consistent with the sizes determined through DLS measurement.

**Figure 3:**
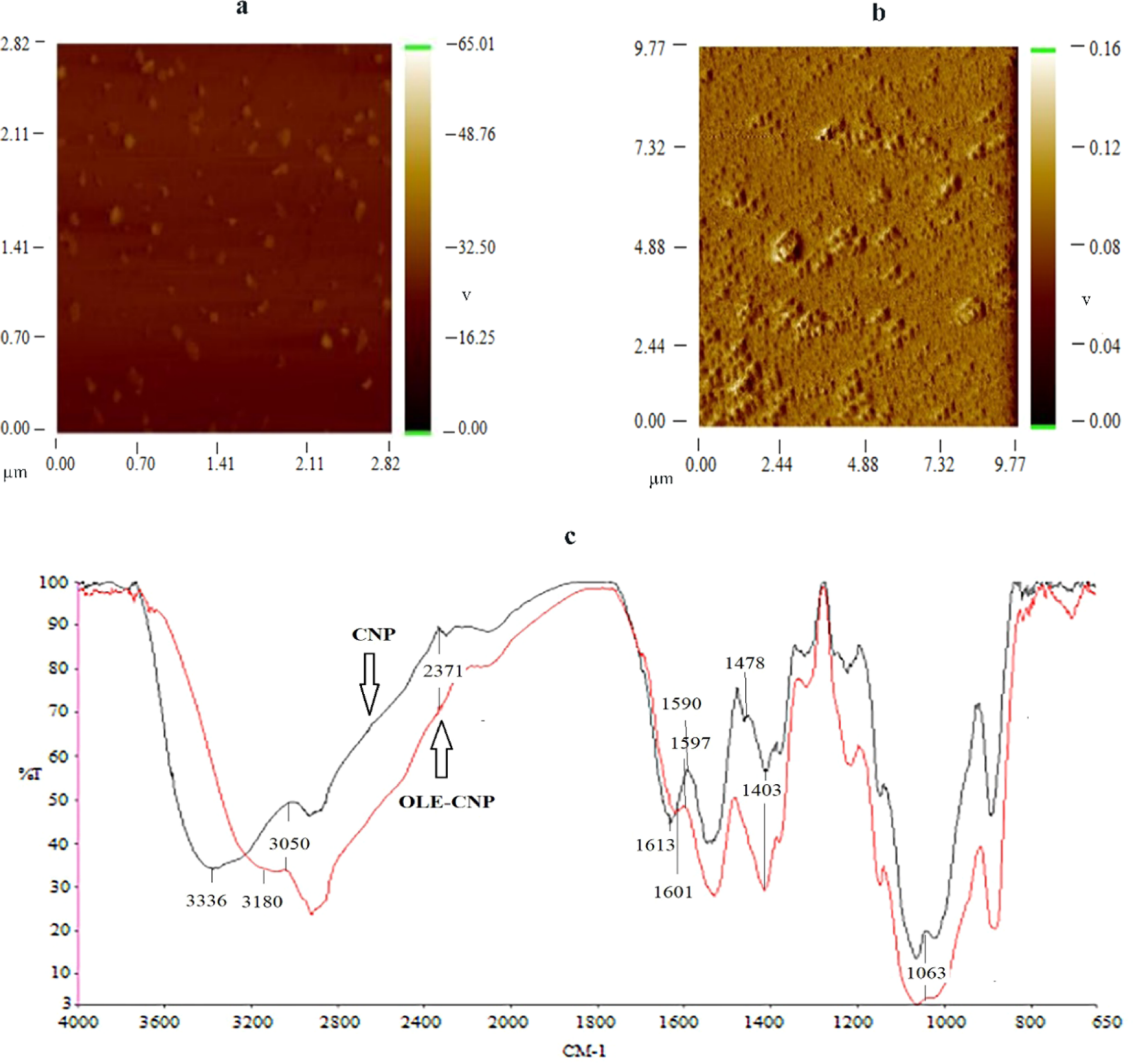
Characterization of OLE-CNPs: (a) AFM image of chitosan nanoparticles; (b) AFM image of OLE-CNPs; and (c) FT-IR spectra of OLE-CNPs and CNPs. The red line represents OLE-CNPs, and the black line represents CNP.

In addition, the morphology of OLE-CNPs was also analyzed using Nanomagnetic Instruments ezAFM on tapping mode. According to AFM images, OLE-CNPs were more spherical than chitosan nanoparticles, lacking pronounced morphological differences (Figure 3a,b). Also, the interactions between OLE and chitosan nanoparticles in OLE-CNPs were analyzed by FT-IR spectroscopy relative to the chitosan nanoparticles. When the spectra of OLE-CNPs were compared with those of CNPs, shifted bands were observed from 3336 to 3180 cm^–1^, and the peak intensity of OLE-CNPs at 3050 cm^–1^ was decreased drastically as a result of the H-bonding that occurs between the OLE and CS matrix. This shifting phenomenon was also attributed to the stretching vibration of −NH_2_ and −OH groups. Furthermore, the band associated with the −NH_2_ group was no longer present, which could be explained by the bonding between the ammonium ions of chitosan and the −OH groups of TPP, as shown in Figure 3c. At a wavenumber of 1478 cm^–1^, a peak corresponding to the C–O stretching bonds unique to CNPs was observed. This linkage between chitosan and TPP may also explain the shifting of the entire bands to lower frequencies when OLE was attached to CNPs.

### 3.3 Effect of OLE-CNPs on Cancer Cell Lines

#### 3.3.1 In Vitro Cytotoxicity Analysis

**Figure 4:**
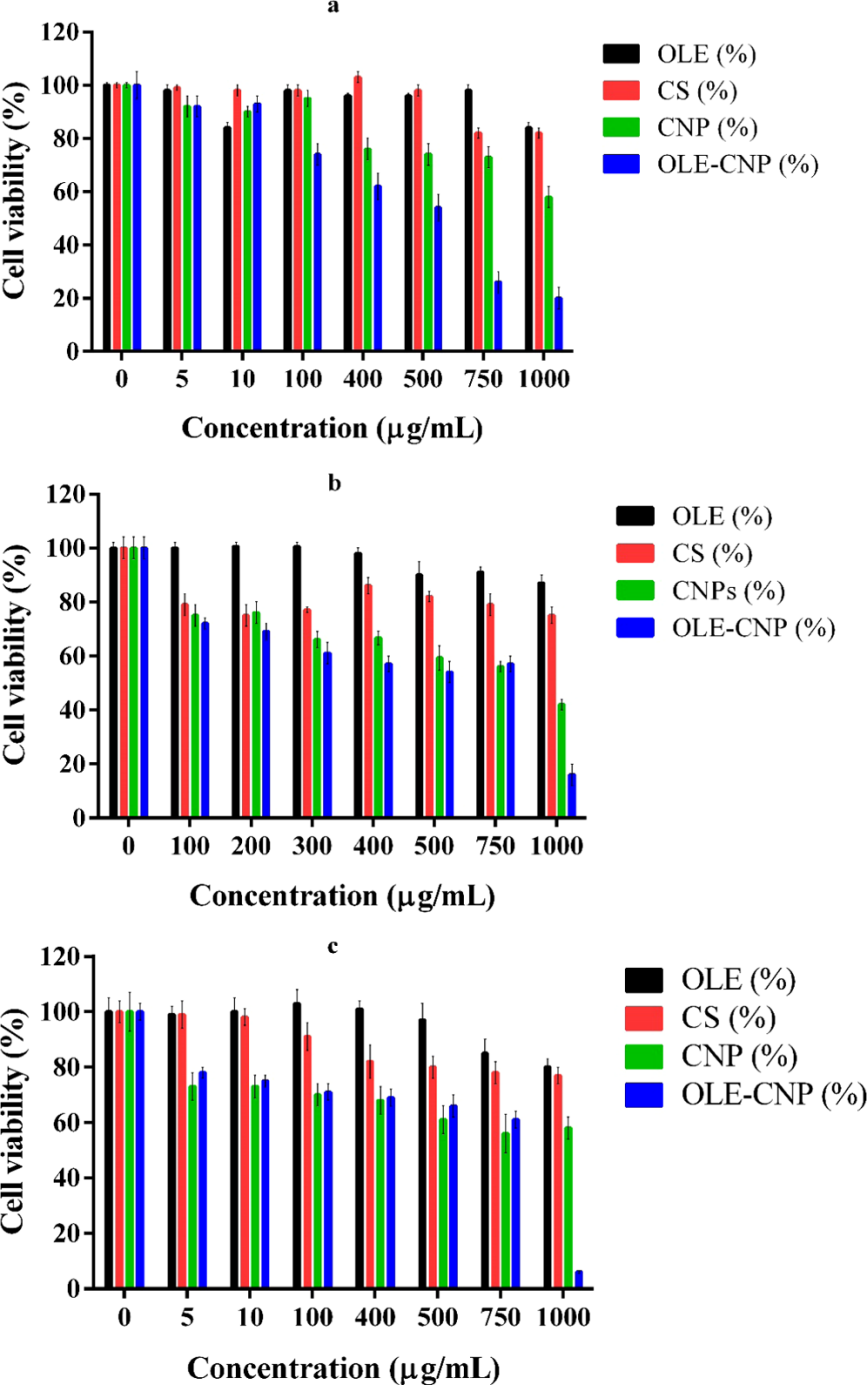
Cytotoxicity of OLE, chitosan (CS), chitosan nanoparticles (CNPs), and OLE-CNPs against (a) A549, (b) MCF-7, and (**c**) BEAS-2B.

Cytotoxicity of various concentrations of OLE-CNPs, CNPs, chitosan, and OLE on two cancer cell lines (A549 and MCF-7) and a healthy cell line (BEAS-2B) was investigated by the MTT assay. The analysis indicated that the cell viabilities of the three cell lines were over 80% against 1000 μg/mL of free OLE (Figure 4). Similarly, the cell viabilities of the three cell lines were over 75% against 1000 μg/mL of chitosan. It was shown that chitosan nanoparticles had more toxic effects on all cell lines, exhibiting viabilities in the range of 42–61%. In addition, 1000 μg/mL of OLE-CNPs considerably reduced the viability of the cell lines to 20% of A549 (Figure 4a), 16% of MCF-7 (Figures 4b), and 5.5% of BEAS-2B (Figure 4c). OLE-CNP appeared to make a significant difference after a certain dose compared to CNP on cancer cell lines. Regarding this, OLE-CNP demonstrated a significantly greater cytotoxic effect on A549 cells and MCF-7, at least 100 μg/mL (Figure 4a) and 1000 μg/mL (Figure 4b), respectively, relative to CNP. Cells exhibited varying sensitivities to OLE-CNP at different concentrations. When the concentration of OLE-CNP is high, BEAS-2B cells demonstrate greater sensitivity to OLE-CNP than cancer cells. Conversely, when the concentration is low, cancer cells exhibit higher sensitivity compared to healthy cells (Figure 4). The IC_50_ of OLE-CNPs was found to be 540 μg/mL for A549 and 810 μg/mL for MCF-7. To the best of our knowledge, there have been no studies on the effect of OLE-immobilized chitosan nanoparticles against A549 and MCF-7. However, there have been studies on the immobilization of leaf extracts of different medicinal plants with chitosan and their effects on cancer cells. Regarding this, the immobilization of the leaf extract of *D. pentandra*, a parasitic plant growing on the mango tree, with chitosan (NPDP) has a high cytotoxic effect on MCF-7 cells, exhibiting about 20% cell viability at 8 mg/mL concentration.^23^ Another recent study showed that *A. squamosa* (named Srikaya, belonging to the Annonaceae family) leaf extract-loaded chitosan (nano-ASLE) has an IC_50_ of 344.48 μg/mL on HeLa cells.^22^ There was a recent study on the effects of OLE immobilization with different support materials on the MCF-7 cell line. For instance, OLE-loaded silver nanoparticles (AgNPs) decreased the cell viability of MCF-7 by 62% at 50 μg/mL.^30^

#### 3.3.2 Cell Cycle Distribution of Cancer Cell Lines against OLE and OLE-CNPs

**Figure 5:**
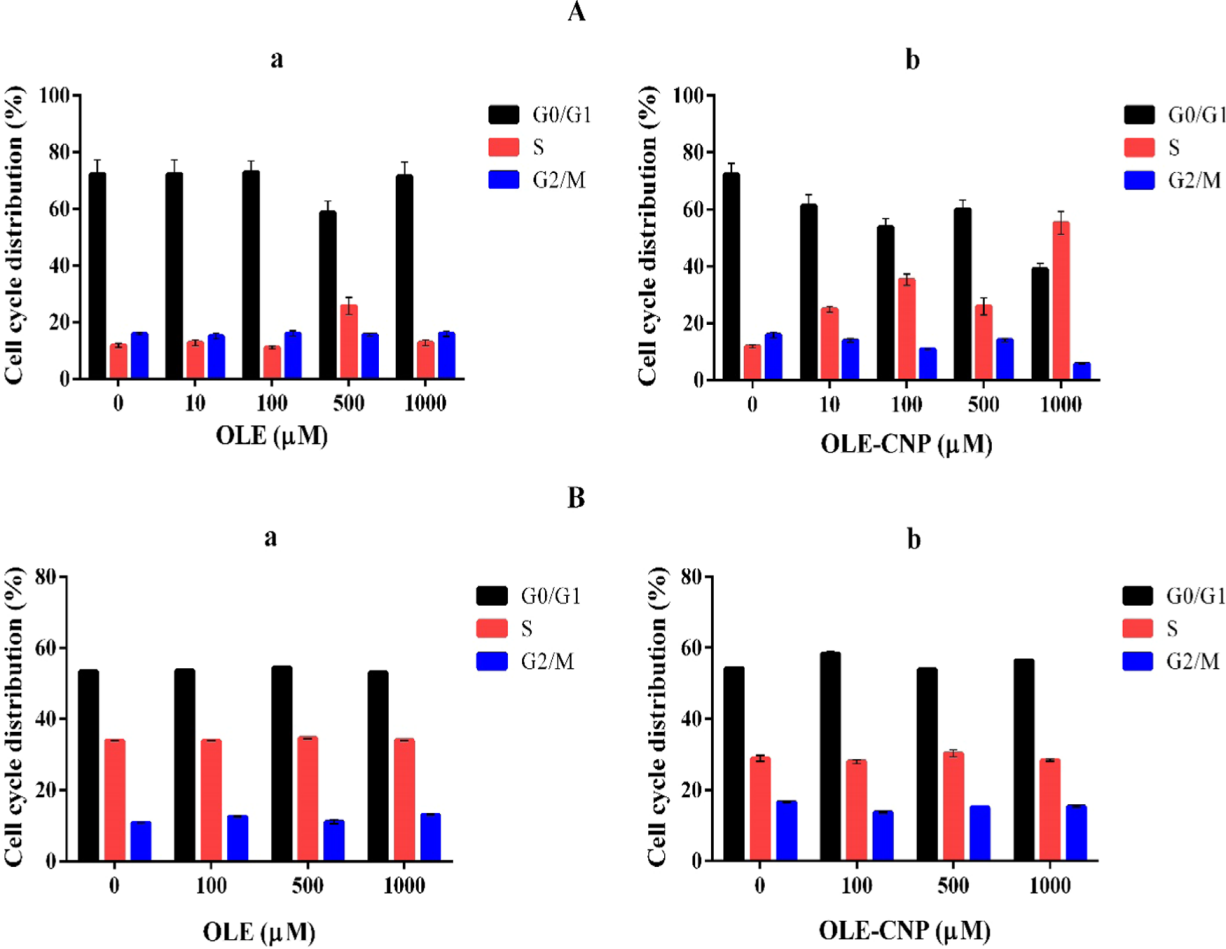
Effects of (a) OLE and (b) OLE-CNPs on cell cycle arrest in (A) A549 and (B) MCF-7 cells.

Cell cycle distribution of A549 and MCF-7 treated with various concentrations (10, 100, 500, and 1000 μM) of free OLE and OLE-CNPs was analyzed by flow cytometry. The analysis indicated that an increase in OLE-CNP concentrations, but not free OLE, gradually decreased the A549 cell number in G2/M arrest from 16 to 5.5%. Similarly, the A549 cell number decreased in G0/G1 from 77 to 40% and increased in the S phase from 12 to 55%, with a gradual increase in OLE-CNPs concentration. Free OLE did not considerably change the cell cycle distribution and exhibited a concentration-independent behavior in A549 cells. The analysis also showed that OLE-CNPs behaved in a dose-dependent manner in all phases (Figure 5A). MCF-7 treated with OLE-CNPs showed a higher percentage of cells in the G2/M phase and a lower percentage of cells in the S phase relative to the free OLE. Free OLE and OLE-CNP treatment exhibited a similar cell percentage in the G0/G1 phase of MCF-7 (Figure 5B). In the literature, *D. pentandra* leaf extract-loaded chitosan (NPDP) nanoparticles induced the G2/M cell cycle arrest in MCF-7 cancer cells.^23^ Similarly, *A. squamosa* leaf extract-loaded chitosan (nano-ASLE) also stimulated the cell cycle arrest in G2/M in WiDr cancer cells.^21^ The present study shows that both cancer cells were predominantly distributed in the G0/G1 phase in the presence of OLE-CNP and free OLE (Figure 5). These results were supported by some studies on MCF-7 cancer cells treated with free OLE or some OLE ingredients (e.g., hydroxytyrosol and oleuropein), showing the cell cycle distribution in G0/G1 arrest.^31^

#### 3.3.3 Apoptotic Influence of OLE-CNPs on Cancer Cell Lines

**Figure 6:**
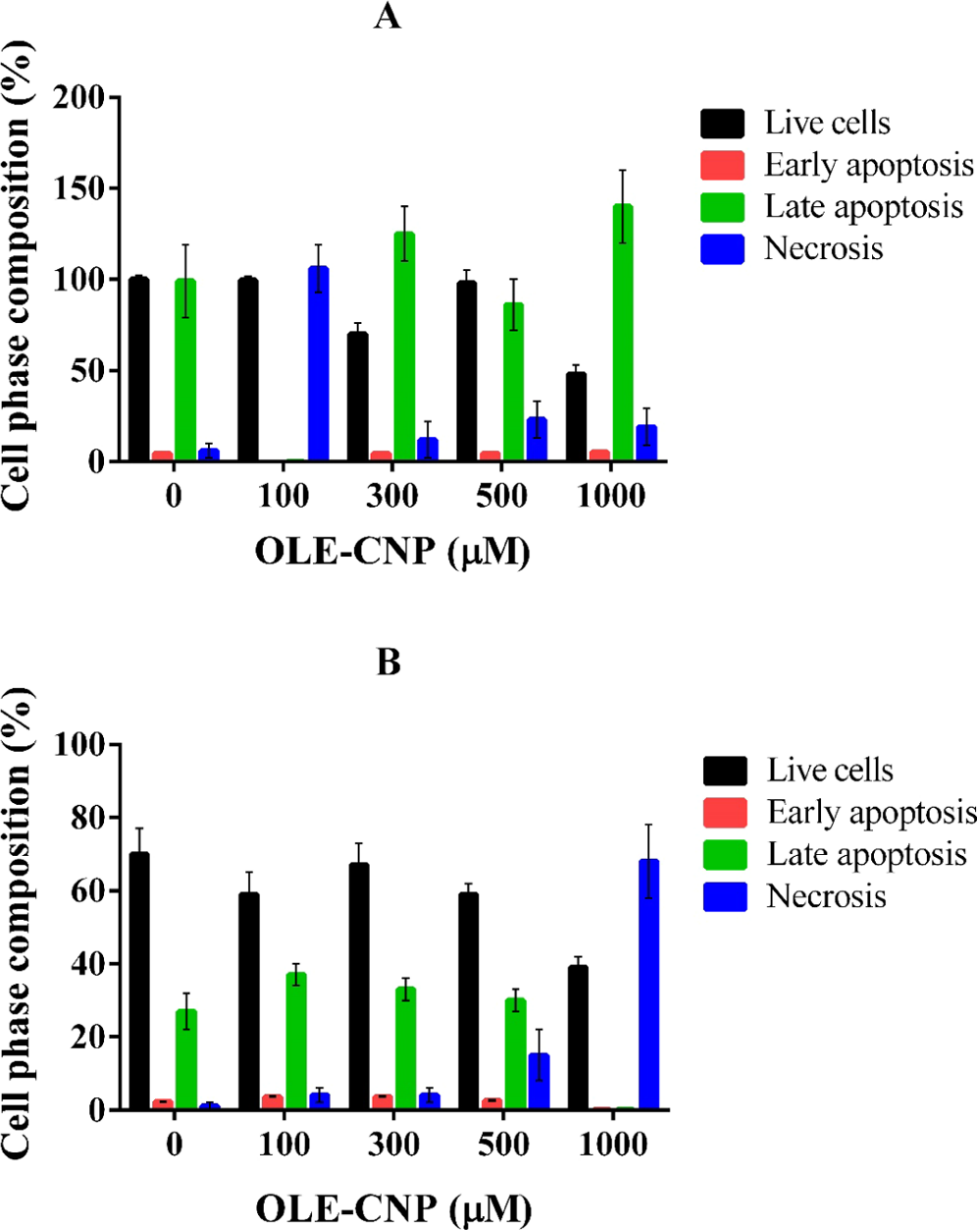
Apoptotic effect of OLE-CNPs on (A) A549 and (B) MCF-7.

The apoptotic influence on A549 and MCF-7 cancer cells treated with different concentrations of OLE-CNPs (50, 100, 300, 500, and 1000 μM), relative to nontreated cells, was analyzed using the Annexin V/propidium iodide (PI) staining method. The analysis showed that the percent cell viability of A549 cells treated with OLE-CNPs decreased compared to that of nontreated cells. In contrast, the percentage of cells in necrosis and late apoptosis phases is enhanced with an increase in the OLE-CNP concentration (Figure 6A). The percentage of viable MCF-7 cells decreased by about 40% at 1000 μM concentration of OLE-CNPs relative to the nontreated cells. Also, the percentage of cells in the necrosis phase did not significantly change up to 500 μM concentration; however, at 1000 μM concentration, the cell percentage increased by 55–70% (Figure 6B). These results suggest that OLE-CNPs strongly induced apoptosis and necrosis in A549 and MCF-7 cells. Anbu et al. (2016) have shown that the leaf extract of the medicinal plant *Gymnema sylvestre* with chitosan nanoparticles led to apoptosis in the human cervical cancer (SiHa) cell line.^32^ In addition, nanochitosan encapsulation of the *Cymbopogon citratus* leaf ethanol extract (NCECC) promoted ROS production, causing apoptosis in human squamous cells (HSC-3).^33^ Another recent study showed that nano-ASLE significantly enhanced caspase-3 expression and caused apoptosis in WiDr cells.^21^

According to optical microscopy analyses, the reduction in A549 and MCF-7 cell numbers was displayed in the presence of OLE-CNPs, relative to other conditions. Shrinking of the cell and formation of apoptotic bodies were seen in the OLE-CNP-treated cells. This result was consistent with the cytotoxicity results of OLE-CNPs on both cells (Figure S2).

## 4 Conclusions

In this study, olive leaves of *O. europaea* were extracted, immobilized, and characterized, and the influence of the extract on breast adenocarcinoma MCF-7 and lung carcinoma A549 cell lines was investigated. The present work is the first report on the effect of olive leaf extract (OLE)-loaded chitosan nanoparticles (CNPs) on breast adenocarcinoma MCF-7 and lung carcinoma A549 cell lines. In this direction, OLE was immobilized on CNPs with 97.5% loading capacity. The CNPs and OLE-CNPs obtained were characterized structurally and functionally in detail by SEM, FT-IR spectroscopy, AFM, and DLS. The results showed that CNPs and OLE-CNPs were homogeneously distributed; the morphology of nanoparticles was spherical, and the average diameter of both nanospheres was nearly 100 nm under optimal conditions. Cytotoxicity results indicated that OLE-CNPs had higher cytotoxic influences on A549 and MCF-7 cell lines compared to free OLE. The IC_50_ of OLE-CNPs on A549 and MCF-7 was found to be 540 and 810 μg/mL, respectively. The assay of cell cycle distribution showed that the above half of the A549 and MCF-7 cells exposed to free OLE and OLE-CNP accumulated in the G0/G1 phase. Apoptosis results demonstrated that OLE-CNPs caused the accumulation of A549 and MCF-7 cells in the late apoptosis and necrosis phases. Based on the results of the present work, OLE-CNPs might be utilized as a supplement in addition to cancer therapy.

## Supporting Information Available

HPLC chromatogram of the olive leaf extract; optical microscopy images of A549 and MCF-7 cells (DOC) (PDF)

## Notes

This article does not contain any studies with human participants or animals performed by any of the authors.
