# Supplementary material for: Immobilization of Olive Leaf Extract with Chitosan Nanoparticles as an Adjunct to Enhance Cytotoxicity
Source: ACS Omega. 2023 Aug 1;8(32):28994–9002. doi: 10.1021/acsomega.3c01494 (PMC10433347; doi:10.1021/acsomega.3c01494)
Supplement: Supplementary file 1 — ao3c01494_si_001.pdf [file ao3c01494_si_001.pdf]

Supporting Information

Immobilization of Olive Leaf Extract with Chitosan Nanoparticles as an Adjunct to Enhanced Cytotoxicity

Burcu Özdamar[1], Yusuf Sürmeli[2,3] and Gülşah Şanlı-Mohamed[1,2*]

1. Department of Chemistry, İzmir Institute of Technology, 35430, İzmir, Turkey
2. Department of Biotechnology and Bioengineering, İzmir Institute of Technology, 35430, İzmir, Turkey
3. Department of Agricultural Biotechnology, Tekirdağ Namık Kemal University, 59030,

   Tekirdağ, Turkey

Corresponding Author

Prof. Dr. Gülşah Şanlı-Mohamed

Izmir Institute of Technology, Science Faculty, Department of Chemistry

Urla, Izmir, Turkey

Phone: +90 2327507515; Fax: +90 2327507509

E-mail: gulsahsanli@iyte.edu.tr, gulsahsanli@gmail.com

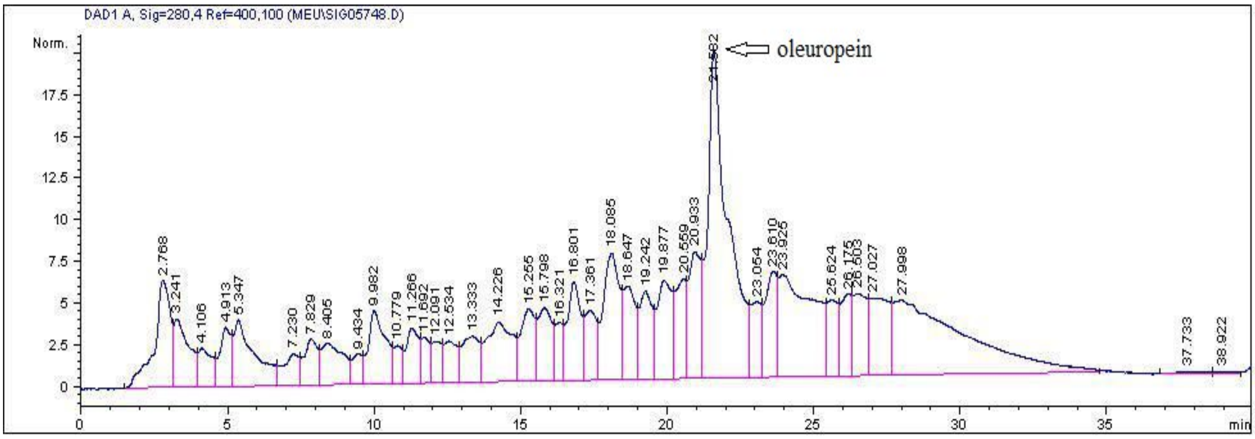

Figure S1. HPLC chromatogram of olive leaf extract

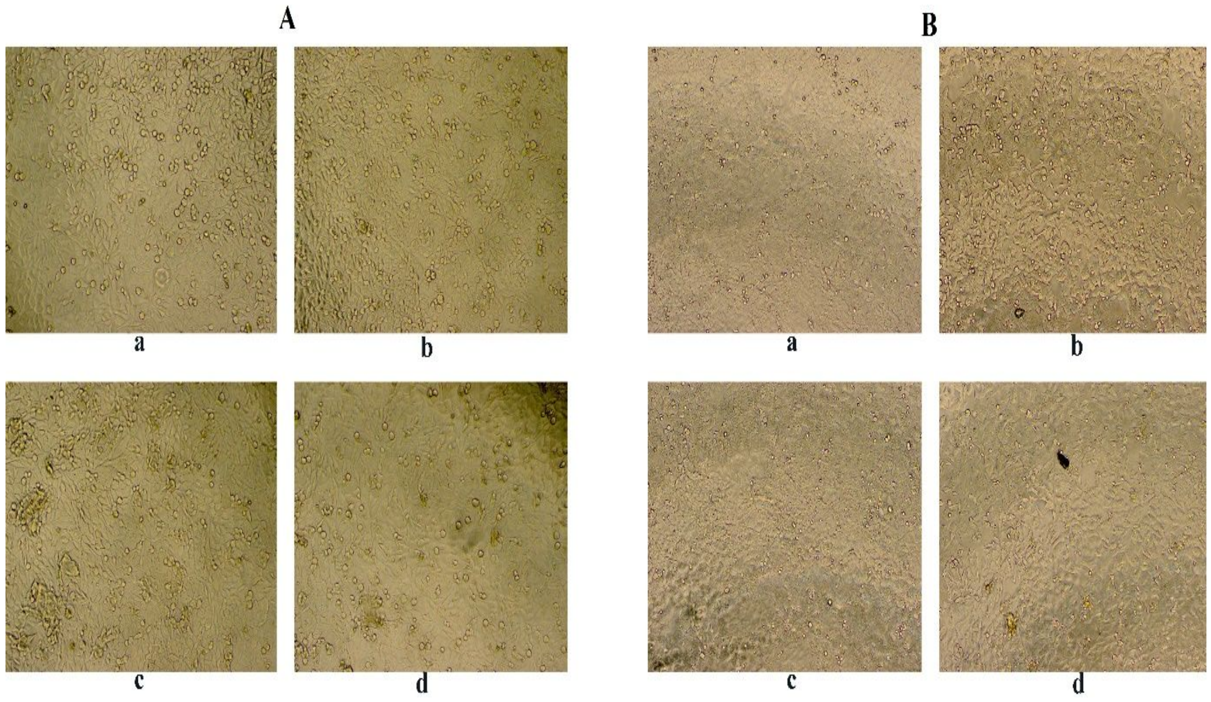

Figure S2. Optical microscopy images of A549 (**A**) and MCF-7 (**B**) cells treated with OLE (**b**), CNPs (**c**), OLE-CNPs (**d**) as well as untreated (control) cell lines (**a**).
